# Transcription Factors as Important Regulators of Changes in Behavior through Domestication of Gray Rats: Quantitative Data from RNA Sequencing

**DOI:** 10.3390/ijms232012269

**Published:** 2022-10-14

**Authors:** Dmitry Oshchepkov, Irina Chadaeva, Rimma Kozhemyakina, Svetlana Shikhevich, Ekaterina Sharypova, Ludmila Savinkova, Natalya V. Klimova, Anton Tsukanov, Victor G. Levitsky, Arcady L. Markel

**Affiliations:** Institute of Cytology and Genetics, 630090 Novosibirsk, Russia

**Keywords:** domestication, molecular mechanisms of behavior, aggressive and tame behavior, midbrain tegmentum, gray rats, neurogenesis, adolescent period, differentially expressed genes, transcription factors, *Ascl3*, RNAseq, motif enrichment analysis

## Abstract

Studies on hereditary fixation of the tame-behavior phenotype during animal domestication remain relevant and important because they are of both basic research and applied significance. In model animals, gray rats *Rattus norvegicus* bred for either an enhancement or reduction in defensive response to humans, for the first time, we used high-throughput RNA sequencing to investigate differential expression of genes in tissue samples from the tegmental region of the midbrain in 2-month-old rats showing either tame or aggressive behavior. A total of 42 differentially expressed genes (DEGs; adjusted *p*-value  <  0.01 and fold-change  >  2) were identified, with 20 upregulated and 22 downregulated genes in the tissue samples from tame rats compared with aggressive rats. Among them, three genes encoding transcription factors (TFs) were detected: *Ascl3* was upregulated, whereas *Fos* and *Fosb* were downregulated in tissue samples from the brains of tame rats brain. Other DEGs were annotated as associated with extracellular matrix components, transporter proteins, the neurotransmitter system, signaling molecules, and immune system proteins. We believe that these DEGs encode proteins that constitute a multifactorial system determining the behavior for which the rats have been artificially selected. We demonstrated that several structural subtypes of E-box motifs—known as binding sites for many developmental TFs of the bHLH class, including the ASCL subfamily of TFs—are enriched in the set of promoters of the DEGs downregulated in the tissue samples of tame rats’. Because ASCL3 may act as a repressor on target genes of other developmental TFs of the bHLH class, we hypothesize that the expression of TF gene *Ascl3* in tame rats indicates longer neurogenesis (as compared to aggressive rats), which is a sign of neoteny and domestication. Thus, our domestication model shows a new function of TF ASCL3: it may play the most important role in behavioral changes in animals.

## 1. Introduction

It is well known that the development of the human civilization is inextricably linked with the domestication of animals. An important factor in the domestication of various species of wild animals has been the transformation of their aggressive behavior toward humans into tolerant and even friendly behavior. Despite a long and successful history of domestication, the genetics of tame and aggressive behavior of domesticated animals toward humans, as well as its involvement in the regulation of these genes, remains unclear.

Modern studies on the transcriptomes of various domestic animal species indicate an influence of a large number of genes on changes in the behavior, coloration, morphology, and physiology of domestic animals [1,2,3,4]. Some of these genes are common among the gene lists published in research articles—usually the genes that are involved in the control of behavior (for example, *Bdnf*, *Grin1*, *Oxt*, and dopamine receptor genes, among others) and various genes that are not related to each other [5,6]. Nonetheless, it is known that behavioral patterns are determined by multiple genes; therefore, one or several genes differentially expressed between tame and wild animals cannot explain the entire spectrum of so-called domestication syndrome [7], which includes alterations in skull morphology [8], coat/feather color [9], the reproductive cycle [10,11,12], and other features. However, the involvement of TF genes in transcriptome changes during the domestication of model animals has not yet been studied.

To determine the molecular mechanisms involved in changing of behavior toward humans in wild animals upon domestication, we used an experimental model: gray rats selected for 45 years for their reaction to humans (either tame or aggressive behavior) at the animal facility of the Institute of Cytology and Genetics, SB RAS (Novosibirsk, Russia). This animal model of domestication is a unique experimental system for analysis of the complex architecture of genetic determination of domestic behavior; in tame and aggressive rats, it is possible to correctly and adequately assess phenotypic manifestations of nonaggressive and aggressive reactions, respectively, upon exposure to a stimulus (for example, an experimenter’s gloved hand, i.e., the glove test) [13,14].

We previously performed genome-wide transcriptomic analysis of samples of the hypothalamus and hippocampus to identify genes differentially expressed between tame and aggressive rats [5,6]; these brain regions belong to the limbic system, which controls emotions [15,16]. The resultant lists of differentially expressed genes (DEGs) were found to contain several TF genes, including *Ascl3* (achaete-scute complex-like 3), which is a member of the basic helix–loop–helix factor class (bHLH) [17]. Typically, members of the bHLH class can form homo- and heterodimers; ASCL3 belongs to the subfamily of achaete-scute-like factors of bHLH TFs, which also includes tissue-specific factors. Members of this subfamily can serve as either activators or repressors [17,18]. TFs of this subfamily participate in neurogenesis, tissue development, and cell differentiation and are critical for proper development of the nervous system [19,20]. Proteins encoded by the genes of this subfamily are directly involved in the segregation of neuroblasts from other epidermal cells; therefore, *Ascl* genes are classified as proneural; the expression of such genes enables ectodermal cells to differentiate into nerve cells [21].

According to the latest data [22], ASCL3 protein belongs to the set of candidate proteins involved in the development of mesencephalic dopaminergic neurons in the midbrain. This brain region contains clusters of serotonergic and dopaminergic neurons and is responsible for inhibitory control of aggressive behavior [23,24,25,26]. The midbrain also includes the periaqueductal gray and substantia nigra. The latter is a group of dopaminergic neurons that form a dopaminergic nucleus. These neurons project to structures of the so-called limbic system, including the previously studied hypothalamus and hippocampus. Therefore, the aim of our work was to determine whether a statistically significant change in gene expression in tame and aggressive rats is a consequence of the differential expression of transcription factor genes, in particular *Ascl3*, which can regulate these genes. Thus, in this work, to elucidate molecular mechanisms underlying the behavioral changes that occur in animals during domestication, we analyzed differences in gene expression profiles between gray rats (*Rattus norvegicus*) selected for either the absence or enhancement of a defensive response to humans (tame and aggressive strains, respectively). We applied two methods: (i) high-throughput RNA sequencing (RNA-seq) analysis of brain samples from the tegmental region of the midbrain (MT) region and (ii) motif enrichment analysis using a newly developed ESDEG tool; these methods helped to investigate coordinated changes in the transcriptome of the animal domestication model.

## 2. Results

### 2.1. Analysis of RNA-Seq Data from Brain Tissue Samples of Tame and Aggressive Rats

Using an Illumina NextSeq 550 system, we sequenced the MT transcriptome of three tame adult male gray rats (*Rattus norvegicus*) and three aggressive rats devoid of any family relationship among them (see Materials and Methods, Section 4.1). This procedure resulted in 182,197,974 reads (each 75 bp in length), which were deposited in the NCBI SRA database (PRJNA668014), as shown in Table 1. Among them, using the pipeline described in Materials and Methods (Section 4.2), we mapped 158,310,590 reads (86.9%) to rat reference genome Rn6 (assembly 2014) (see Table 1). Then, we identified 14,039 genes expressed within the MT of rats under our experimental conditions (Table 1). To minimize the false-positive error rates, we also applied Benjamini–Hochberg correction for multiple comparisons, resulting in 42 DEGs within the MT of tame rats versus aggressive rats (Table 1).

These DEGs are listed in Table 2. In particular, the *Ascl3* gene encoding achaete-scute complex-like transcription factor 3 showed the highest significance of enrichment (P_ADJ_ = 3.16 × 10^−4^) and a log_2_ (fold change) of −2.82, ranking fifth among all upregulated DEGs in the tame rat samples (fold change or fold is a ratio of an expression level of a given gene in tame rats to that in aggressive rats, hereafter referred to as log_2_ fold; see Table 2).

Thus, we obtained a list of differentially expressed genes between tame and aggressive rats in the MT (42 genes in total; Table 2), among which genes encoding three TFs (ASCL3, FOS, and FOSB) were identified, in addition to ARC (known as the master regulator of synaptic function and of numerous neuronal signaling pathways), extracellular-matrix components (HSPA1A, HSPA1B, TAC3, and VIP), transporter protein (SLC4A5), neurotransmitter (HCRT), signaling molecules (serotonin receptors HTR3A and HTR5B, LILRB3L, and RETSAT), and immune system proteins (DEFB17 and VIP). The observed significant differences in the expression levels of these genes between tame and aggressive rats indicate major biochemical and physiological rearrangements in the activity of neurons at the metabolic level, along with changes in behavior. The proteins encoded by the DEGs differ both in terms of their functions and in the neurotransmitter systems with which they are affiliated; this state of affairs results in a multifactorial system determining the trait for which our rats were artificially selected.

Our list of DEGs was functionally annotated in the David Bioinformatics Resources database [27]. The only category statistically significantly (*p* = 0.0089) enriched in this gene set was a group of genes united by the term “Signal” (UniProt Category Domain, KW-0732): *Htr3a*, *Fcgr2b*, *Lypd3*, *Cthrc1*, *Col15a1*, *Defb17*, *Hcrt*, *Lilrb3l*, *Mpeg1*, *Olfml1*, *Pcdhga1*, *Retsat*, *Spint1*, *Tac3*, and *Vip*, in agreement with the current understanding of the relation of signaling pathways with behavioral response. The absence of significant enrichment with more specific terms can be explained by the multifactorial nature of behavioral responses and of the evolutionary process.

### 2.2. Gene Expression Analysis in the MT of Tame and Aggressive Rats by qPCR

**Figure 1:**
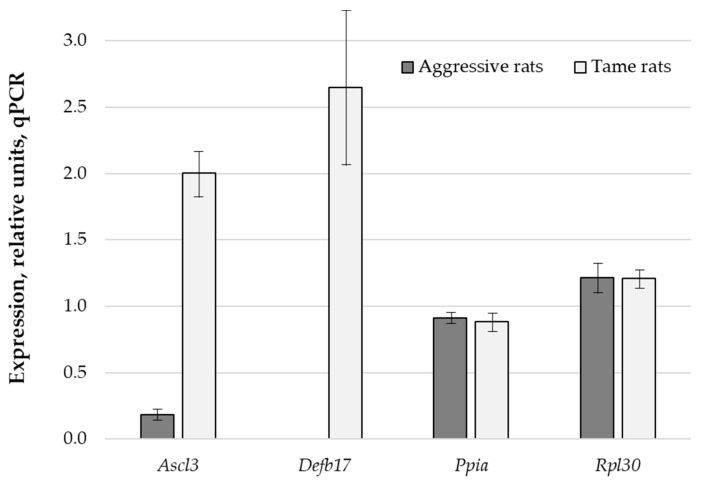
Normalized mRNA gene levels in the midbrain tissues of tame rats (white bars) versus aggressive rats (gray bars) for two assays (*Ascl3* and *Defb17*) and two reference (*Ppia* and *Rpl30*) genes. Genes *Ascl3* and *Defb17* are statistically significantly overexpressed in the MT. The bar heights denote mean values, error bars represent standard error, and asterisks denote statistical significance (*p* < 0.05) according to both the Mann–Whitney *U* test and Fisher’s Z-test.

The differential gene expression results obtained in the RNA-seq experiment (Table 1) were experimentally validated by qPCR (in the MT samples of eight tame and eight aggressive rats from the 90th generation of selection) on two selected genes—*Ascl3* (achaete-scute complex-like transcription factor 3) and *Defb17* (defensin beta 17)—as well as two reference genes: *Ppia* and *Rpl30*. The results of the RNA-seq analysis were successfully confirmed; expression levels of genes *Ascl3* and *Defb17* were significantly higher in the tame rats than in aggressive rats (*t*-test, *p* < 5 × 10^−6^, Figure 1). Expression results for the reference genes did not differ significantly between the tame and aggressive rats according to qPCR analysis.

### 2.3. Analysis of Enrichment with Binding Sites for TFs

**Figure 2:**
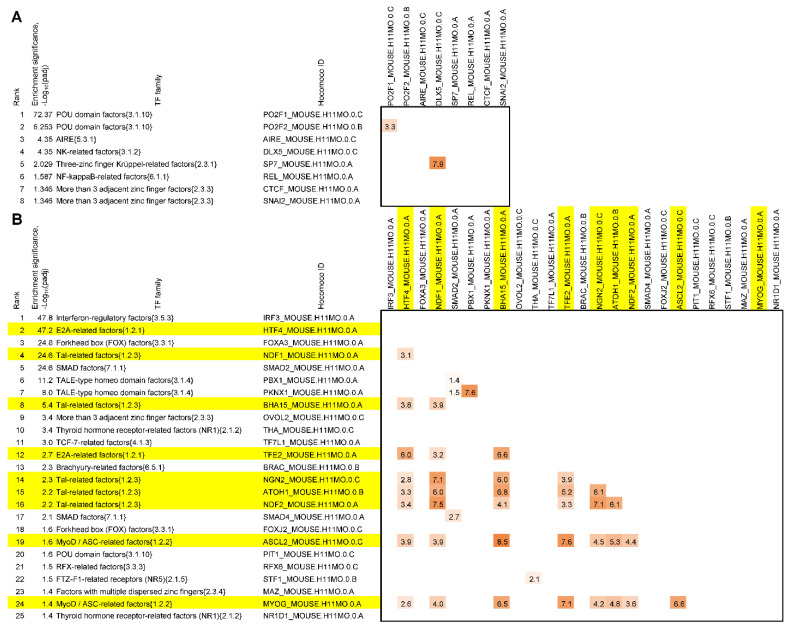
Ranking and classification of the overrepresented motifs for known TFs in the promoters of DEGs selected according to criteria P_ADJ_  <  0.01 and log_2_ fold > 1. Panels (**A**,**B**) show 8/25 top-scoring motifs detected for the promoters of genes upregulated and downregulated in the brain tissue samples of tame rats, respectively (see Table 2). Motif overrepresentation was estimated by the ESDEG tool (see Materials and Methods, Section 4.4). Motifs of known TFs were compiled from the murine core collection of the Hocomoco database (see Materials and Methods). On the left, each panel shows four columns, indicating the ranks of motifs, the significance of their enrichment computed by ESDEG [(log_10_(p_adj_)], the respective TF families according to the classification of murine TFs based on their DNA-binding domains [17], and the names of TFs (Hocomoco ID). The yellow color in panel B indicates motifs for TFs from the bHLH class belonging to three closely related families (E2A-related factors {1.2.1}, MyoD/ASC-related factors {1.2.2}, and Tal-related factors {1.2.3}) possessing a similar E-box binding motif. On the right, each panel presents the bottom left corner of the symmetrical matrix, indicating pairwise similarities of motifs as estimated by the permutation test described earlier [29]. Empty cells imply significantly distinct motifs (*p*-value > 0.05), the shades from light orange to dark orange denote significantly similar motifs (*p*-value < 0.05), and the numbers indicate the logarithm of pairwise significance: (log_10_(*p*-value).

Above, we revealed that TF genes *Ascl3* and *Fos*/*Fosb* were significantly upregulated and downregulated, respectively, in the brain tissue samples of tame rats (Table 2). These TFs are included in the standard classification analysis of murine TFs [17]. Fos-related factors {1.1.2} (hereafter, numbers in curly brackets correspond to the classification of murine TFs based on their DNA-binding domains [17]) and MyoD/ASC-related factors {1.2.2}—respective TF families for these factors—possess well-known motifs in the publicly available Hocomoco library of motifs, which is based on a massive analysis of ChIP-seq data [28]. Therefore, we were able to test the overrepresentation of motifs of known TFs in the promoters of the DEGs. The standard methods for motif enrichment analysis utilizing the exact Fisher’s test appeared to be ineffective due to the small sizes of our promoter datasets (20 and 22 DEGs; see Table 2). Accordingly, we developed an original method called ESDEG to test the overrepresentation of motifs independent of dataset size (see Section 4.4, Materials and Methods). We applied ESDEG to the promoter sets of the genes upregulated and downregulated in the brain tissue samples of tame rats (Table 2), employing the library of motifs for known murine TFs (see Section 4.4, Materials and Methods). The names of the respective TF families were compiled based on their DNA-binding domains [17] for enriched motifs to simplify the interpretation of the results. Additionally, we performed a permutation test [29] to estimate the significance of pairwise similarity between the motifs that were found to be enriched in each set. The resulting tables of overrepresented motifs for known TFs detected in promoters of the genes upregulated and downregulated in the brain tissue samples of tame rats are shown in Figure 2A,B, respectively. The list of 25 motifs corresponds to the set of promoters of the genes downregulated in the brain tissue samples of tame rats, whereas the other list, corresponding to the set of promoters of the genes upregulated in the brain tissue samples of tame rats, contains only eight motifs. The IRF3 motif ranks first in the motif enrichment list for the genes downregulated in the brain tissue samples of tame rats. Because the *Irf6* gene showed an upregulation trend in the RNA-seq experiment (although it does not fulfill the DEG criteria: p_adj_ = 0.051 < 0.1, log_2_ fold = (0.87, see Table S1), we wondered whether it could be responsible for the enrichment of the IRF3 motif. We found that the IRF6 motif does not possess a significant similarity to the rest of the TFs that are members of the interferon-regulatory factor family {3.5.3} (*p* > 0.05, [29]); thus, the relation of slightly upregulated IRF6 TF and IRF3 motif enrichment seems insignificant. A large proportion of the motif enrichment list for genes downregulated in the brain tissue samples of tame rats (9 of 25) are motifs for TFs of closely related families: E2A-related factors {1.2.1}, MyoD/ASC-related factors {1.2.2}, and Tal-related factors {1.2.3}. All the mentioned TF families belong to the bHLH class. The motifs of TFs from these families are very similar and are known as E boxes. These motifs showed a highly significant mutual similarity (Figure 2A, 0.01 < (log_10_(*p*-value) < 1 × 10^−9^), coinciding with the significant upregulation of the *Ascl3* gene in the brain tissue samples of tame rats (P_ADJ_ = 3.16 × 10^−4^ and log_2_ fold = 2.82, see Table 2). A recent large all-against-all benchmarking of positional weight matrix models [30] was used for recognition of motifs. It was demonstrated here that the best-performing positional weight matrix for a given TF is often related to another TF, usually from the same family. Thus, we propose that the overrepresented bHLH motifs found in promoters of the genes downregulated in the brain tissue samples of tame rats match potential binding sites for ASCL3 TF with related partner TFs from the same bHLH class. Thus, we compiled a sizeable list of ASCL3 targets among genes downregulated in the brain tissue samples of tame rats; this is consistent with the fact that the ASCL3 TF can act as a repressor toward positively acting bHLH TFs [31]. We did not find any other TF genes in the lists of DEGs (Table 2) corresponding to other enriched motifs (Figure 2).

We noticed very significant enrichment with motifs for TFs from the POU domain factor family {3.1.10} for promoters of the genes upregulated in the brain tissue samples of tame rats. Although we did not detect any differential expression for any gene from the POU domain factor family, this finding is worth a mention because the TFs from this family regulate a wide range of developmental processes—from specification of the early embryo to terminal differentiation. Moreover, the most enriched motif in this group belongs to the POU2F2 TF, which is known to be a regulator of neuronal development and differentiation [32]. We suggest that this observation deserves further investigation. Furthermore, we noted that the milder threshold for the fold ratio (P_ADJ_ < 0.1, fold change > 1.5 or log_2_ fold > 0.585) abrogates the enrichment of motifs for bHLH TFs in the promoters of this extended list of DEGs (see Table S1) regarded as downregulated in the brain tissue samples of tame rats at this threshold, although it revealed the FOSL2_MOUSE.H11MO.0.A motif from the Hocomoco database in the motif enrichment list for the promoter set of the genes regarded as upregulated in the brain tissue samples of tame rats at this threshold (see Figure S1). Consequently, we believe that this motif enrichment may be consistent with the finding that FOS/FOSB TFs are downregulated in the brain tissue samples of tame rats (Table 2).

**Figure 3:**
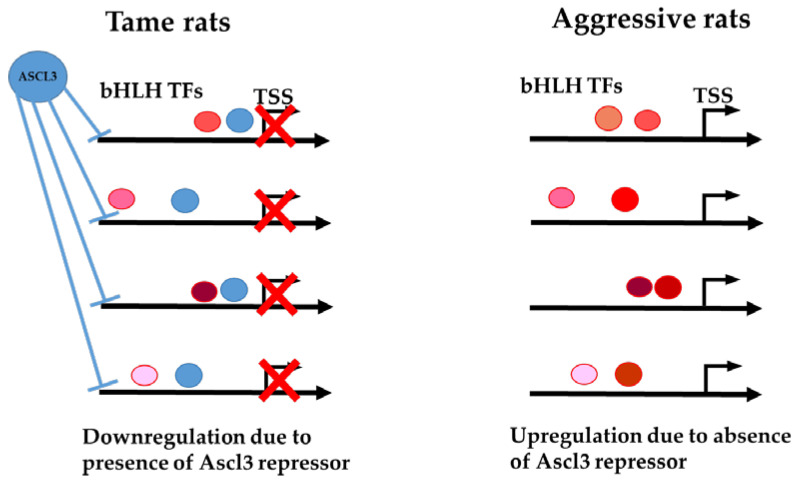
Proposed molecular mechanism for changes in gene expression between tame and aggressive rats due to differences in ASCL3 expression and its involvement in the regulation of these genes. TFs of the bHLH class acting as transcription activators are shown in red/pink/orange. Blue color shows TF ASCL3 acting as a repressor for target genes of various TFs of the bHLH class.

In conclusion, we identified a correspondence between the overexpression of TF gene *Ascl3* in tame rats and changes in the expression of genes in the list of DEGs; TF gene *Ascl3* is one of the top-scoring genes in the list of DEGs upregulated in the brain tissue samples of tame rats (Table 2). Additionally, we detected that the motifs matching potential binding sites for TF ASCL3 and significantly similar motifs for its putative partner TFs from the same bHLH class constitute a large proportion (9 of 25; Figure 2) of all motifs significantly enriched in the set of the genes downregulated in tame rats. Furthermore, Yoshida et al. [31] previously proved not only the possibility of an interaction between ASCL3 and another bHLH TF, MYOD1, but also that in vitro, ASCL3 acts as a repressor for target genes of other bHLH TFs that enhance transcription. Considering these facts together, we can conclude that ASCL3 is involved in the regulation of the set of the genes downregulated in tame rats, acting as a repressor. Moreover, the large number and variety of bHLH TF binding motifs enriched in the set of genes downregulated in tame rats (Figure 2B) suggest that ASCL3 may repress target genes of various TFs of the bHLH class. The proposed molecular mechanism for changes in gene expression between the tame and aggressive rats due to differences in ASCL3 expression is shown in Figure 3.

## 3. Discussion

### 3.1. Genes Encoding Transcription Factors Ascl3, Fos, and Fosb and Gene of Transcription Regulator Arc

Below we consider TF genes *Ascl3*, *Fos*, and *Fosb* and the gene of a transcription regulator, *Arc*, in detail. The presence of these four genes among the DEGs in the rats selected for their reaction to humans is of considerable importance because the products of these genes, by definition, regulate the expression of a considerable number of genes. Significantly upregulated TFs in aggressive rats include two genes, *Fos* and *Fosb*, which are fairly well studied, as well as a gene of an important transcription regulator, *Arc*. They belong to the group of immediate early genes and are well-known markers of neuron activation [33,34,35,36]. Tight regulation of the expression of immediate early genes is crucial for synaptic plasticity, learning, and memory [37,38]. At baseline, these genes are expressed weakly. Biological activities within neurons lead to a rapid increase in their expression and a subsequent return to baseline levels within a few hours [39]. Nevertheless, what exactly leads to the rapid upregulation of these genes is still unclear; immediate early genes, in particular *Fos*, *Fosb*, and *Arc*, show different temporal patterns of activation after exposure to stress [40,41]. FOSB is a key TF causing changes in gene expression in medium spiny neurons of the nucleus accumbens; this group of neurons in the ventral striatum is an important component of the mesolimbic pathway involved in the reward system and in the formation of pleasure, laughter, addiction, aggression, fear, and the placebo effect [42]. The *Arc* gene does not encode a classic TF but can localize to the nucleus, thereby regulating gene transcription [43], is involved in various neuronal signaling pathways [44,45], and controls network stability [46]. *Arc* is a conserved gene in vertebrates [47] and is predominantly expressed in glutamatergic neurons of the cortex and hippocampus. ARC is the main regulator of the expression of a substantial number of genes, thus controlling mRNA levels of more than 1900 genes involved in neuronal plasticity and intrinsic excitability, including many TFs [36].

We investigated the next level of possible regulation of TFs owing to *Arc* downregulation in the brain tissue samples of tame rats (Table 2) and its relation to the results of motif enrichment analysis (Figure 2). For instance, Leung et al. [36] have provided a list of 40 transcriptional regulators or TFs, the mRNA levels of which are altered when activity-dependent *Arc* expression is prevented. These proteins are of neuronal relevance and were designated as direct targets for the ARC protein in [36]. The Hocomoco database [28] indicates that only 11 of these 40 transcriptional regulators are well-studied TFs because they possess the motif models in the murine core collection. We next performed a detailed analysis of 6 of these 11 TFs found to be direct targets of ARC: *Atf3*, *Fosb*, *Jun*, *Neurod2* (*Ndf2*), *Nfil3,* and *Pou2f2*. Notably, all of them, except *Pou2f2*, proved to be activated by ARC, whereas *Pou2f2* is repressed [36]. The POU2F2 TF belongs to the POU2 (Oct-1/2-like factor) family {3.1.10.2}; this TF is underexpressed and has an intermediate expression pattern between tissue-specific and ubiquitous [48]. POU2F2 regulates the distribution of neurons and inhibits gene expression in neuronal cells [32]. Motifs for POU2F2 and for its closest homolog, TF POU2F1, are similar (*p*-value < 1 × 10^−3^, [29]); these are the top-two ranking motifs in the list of enriched motifs for the genes upregulated in the brain tissue samples of tame rats (Figure 2A, ESDEG tool, (log_10_(p_adj_) > 72 and (log_10_(p_adj_) > 6). 

Although we failed to detect differential expression of the *Pou2f2* gene, the known fact that the ARC protein [36], the gene of which is included in the list of repressed genes in tame rats (Table 2), can repress the *Pou2f2* gene may explain the significant enrichment of Pou2f1/Pou2f2 motifs in upregulated genes in tame rats. We suggest that this observation deserves further investigation.

The FOSB TF belongs to the Fos-related factor family {1.1.2}. TFs from this family can dimerize with TFs from the closely related family of Jun-related factors {1.1.1}. TFs from the JUN and FOS families form the TF complex known as AP-1, and FOSB regulates cell proliferation, differentiation, and transformation [49]. Two members of the JUN and FOS families, TFs JUN and ATF3, were also detected in the list of 11 direct targets of ARC [36] and are annotated in the Hocomoco database. FOSB, JUN, and ATF3 are known to be expressed ubiquitously [48]. Among these three TF genes under study, only *Fosb* was significantly downregulated in the brain tissue samples of tame rats (Table 2), and we noted that the FOSL2_MOUSE.H11MO.0.A motif, which shares a high similarity with murine motifs for FOSB, JUN, and ATF3 (*p*-value < 1 × 10^−9^, [29]), is enriched in the promoter set of the genes regarded as upregulated in the brain tissue samples of tame rats at the mild threshold (fold change > 1.5, Figure S1). *Neurod2* (NDF2) is a highly tissue-specific TF that is expressed only brain tissue [48] and belongs to the Tal-related factor family {1.2.3} of the bHLH TF class. This TF is implicated in neuronal determination and is essential for the repression of the genetic program for neuronal differentiation. Although NDF2 is not differentially expressed here, motifs specific to this TF are enriched in the list of promoters of the genes downregulated in the MT tissue samples of tame rats (see Figure 2B). NFIL3 is a ubiquitously expressed TF [48] from the C/EBP-related family {1.1.8}; it almost satisfied the criteria for upregulated DEGs in tame rats (P_ADJ_ = 0.085 < 0.1 and log_2_ fold = 0.71 < 1, i.e., fold change = 1.64, see Table S1). In conclusion, we documented significant enrichment with DNA motifs specific to a number of TFs, the genes of which are direct targets of ARC, but failed to detect their differential expression. The latter result may be due to the highly dynamic nature of immediate early genes and may become a subject of our further research.

### 3.2. The Effect of the SNP in the TATA Box of the Ascl3 Gene

In tame rats compared to aggressive rats, the *Ascl3* gene is statistically significantly upregulated not only in the MT but also in the hypothalamus [5] and hippocampus [6], as well as in the periaqueductal gray (our unpublished data). We analyzed the initial stage of human ASCL3 gene transcription, which is initiated by the interaction of TATA-binding protein (TBP) with the gene promoter [50]. In version 151 of the dbSNP database [51], 18 single-nucleotide polymorphisms (SNPs) were found in the promoter region within the potential TBP-binding site. Using the SNP_TATA_Z-tester Web service [52], we made a prediction about the change in gene expression (as presented in refs. [53,54,55,56,57,58,59,60,61,62,63,64,65,66,67]; see Supplementary Materials, Figure S2 and Table S2). Among the 18 SNPs, two candidates were identified that statistically significantly change the affinity of TBP for this promoter, as described in detail in our previous work [6]. We tested this prediction for one SNP marker via an electrophoretic mobility shift assay (Supplementary Materials, Figure S3). An in vitro experiment regarding the influence of this SNP on the TBP–DNA interaction revealed that for the human *ASCL3* gene, the affinity of TBP weakens by 40% (K_D_ = 12 and 17 nM, respectively) as a result of the T/c substitution (rs1049743008, where T and c are the major and minor alleles, respectively) in the potential TBP-binding site. The Supplementary Materials include ASCL3 electropherograms that illustrate this finding. We can conclude that normal expression of the ASCL3 gene for humans corresponds to overexpression of this gene in tame rats, whereas the minor allele corresponds to aggressive rats, which is consistent with the mainstream understanding of the role of self-domestication in human evolution [68].

### 3.3. ASCL3 as an Important Regulator of Changes in Behavior through Domestication of Gray Rats

As mentioned above, the bHLH class is widely involved in cell differentiation and tissue development, including neural cell development and differentiation [18]. Our results show that ASCL3 has a major impact on these processes by reducing the expression of these genes in tame rats compared to aggressive rats.

The most important representative of the bHLH class of TFs regulating neural development is the achaete-scute complex-like (ASCL) transcription factor. This observation makes the latter proteins prime candidates for TFs, the target genes of which can be repressed by TF ASCL3. ASCL TFs are directly involved in neural development and neurogenesis [19,69]. In particular, TF ASCL1 is transiently expressed during nervous system development (including the development of the sense of smell and the autonomic nervous system [20]) and controls early and late phases of neurogenesis, the division of radial glia progenitor cells, and the migration of postmitotic neurons [70,71]. Under certain conditions, *Ascl1* overexpression can cause dedifferentiation of adult hippocampal neurons into oligodendrocytes [72] and effectively induce the conversion of postnatal astrocytes into functional synapse-forming neurons in the dorsal midbrain of mice [73]. TF ASCL2 is specifically expressed in neuronal progenitors [69,70], whereas ASCL4 is thought to determine the differentiation pathway of nerve and epidermal cells by acting as a proneural protein [74].

Neurogenesis, as a process of differentiation of a neuronal progenitor and its integration into a neuronal network, is most active in the prenatal period; however, it continues in the adult organism, albeit less intensively [75]. In adult mammals, new neurons constantly form in two brain regions: the subgranular zone of the hippocampus and the subependymal zone of lateral ventricles. Therefore, the overexpression of the *Ascl3* gene in the hippocampus of tame rats [6] could be expected based on our assumptions, although its significant underexpression in aggressive animals is difficult to interpret. In this work, we detected *Ascl3* upregulation in the MT, raising the possibility of neurogenesis in the midbrain. Data reported in the literature on this subject are rather contradictory [76,77,78] and do not provide a definitive answer. Neurogenesis in the MT region, just as in other non-neurogenic areas of the brain, probably indicates immaturity of the organism and ongoing formation of neurons.

Therefore, we can theorize that in tame rats, neurogenesis is active for a longer period, continuing into the second month of life. On the other hand, TF ASCL3, by repressing the expression of genes that are responsible for neurogenesis, affects the rate of maturation. Accordingly, the genes whose expression levels change under the influence of TF ASCL3 become differentially expressed and determine the phenotype of tame behavior.

If our theory about prolonged activity of neurogenesis in 2-month-old tame rats owing to *Ascl3* overexpression is correct, then it is possible that the puberty period in these animals is also delayed compared to that in aggressive rats because the neuroendocrine system directly controls the reproductive function. A study on the effect of long-term selection for behavior on the reproductive system activity of tame and aggressive rats revealed that the testosterone level and relative weights of the testes and testicular appendages are significantly lower in male tame rats than in aggressive rats at 2 months of age [79]. In this regard, the positive correlation between aggressiveness and testosterone levels is relevant, the latter being significantly higher in 2-month-old aggressive male gray rats in comparison with not only tame males but also unselected males [79].

It is believed that delayed neurogenesis is an indicator of neoteny [80]. In tame animals, the maturation period is longer than in wild animals; for example, in a research article on another model animal, the silver fox, it was demonstrated that prolonged artificial selection leads to increased neurogenesis in the hippocampus of tame foxes and that this process is inversely proportional to increased aggressiveness toward humans [81]. Taken together, the results with respect to prolonged neurogenesis and delayed puberty in tame rats (which are often used as an experimental model of behavior of domesticated animals) suggest that the manifestation of neoteny is a characteristic trait of animals during domestication [82,83,84,85].

Therefore, in tame and aggressive rats, a new function of TF ASCL3 was demonstrated; by changing the expression of gene networks, ASCL3 may play an important role in changes in the behavior of animals during domestication.

## 4. Materials and Methods

### 4.1. Experimental Animals

This study was conducted on adult male gray rats (*R. norvegicus*) artificially bred for more than 90 generations for either aggressive or tame behavior (as two outbred strains). The rats were kept under standard conditions at the Conventional Animal Facility at the ICG SB RAS (Novosibirsk, Russia) as described elsewhere [14,86,87]. A total of 22 rats (11 aggressive and 11 tame) were included in this study, each 2 months old and weighing 250–270 g, all from different unrelated litters. All the rats were decapitated. Using a handbook technique [88], we excised samples of the MT, which were then flash-frozen in liquid nitrogen and stored at −70 °C until use. Every effort was made to minimize the number of animals under study and to prevent their suffering. This work was conducted in accordance with the guidelines of the Declaration of Helsinki, Directive 2010/63/EU of the European Parliament, and of the European Council resolution of 22 September 2010.

The research protocol was approved by the Interinstitutional Commission on Bioethics at the ICG SB RAS, Novosibirsk, Russia (approval documentation No. 97 dated October 2021).

### 4.2. Total RNA Extraction, Library Construction, and RNA-Seq Data Analysis

Library preparation, sequencing, and bioinformatics manipulations can be found elsewhere [5,6]. We used the following criteria to determine whether a gene was differentially expressed: statistical significance at a *p*-value of <0.1 adjusted for multiple comparisons and a fold change of gene expression of >2.0 (or a fold change of >1.5).

### 4.3. qPCR

To selectively and independently verify the tame-versus-aggressive rat hippocampal DEGs found in the present study (Table 2), we performed a qPCR control assay on the total RNA extracted only from the remaining samples of the hypothalamus of tame (n = 8) and aggressive (n = 8) rats. First, with the help of TRIzol™, we isolated total RNA, purified it on Agencourt RNAClean XP Kit magnetic beads (Beckman, #A63987), and quantified it by means of a Qubit™ 2.0 fluorometer (Invitrogen/Life Technologies), along with a high-sensitivity RNA kit (Invitrogen, cat. #Q32852). Then, we synthesized cDNA using a reverse transcription kit (Syntol, #OT-1). Next, using the PrimerBLAST web service [89], we designed oligonucleotide primers for qPCR (Table 3). Then, we carried out qPCR on a LightCycler^®^ 96 (Roche, Basel, Basel-Stadt, Switzerland) with an EVA Green I kit in three technical replicates. We determined qPCR efficiency by means of serial cDNA dilutions (standards). In line with the commonly accepted recommendations [90], we simultaneously analyzed two reference genes, namely *Ppia* (peptidylprolyl isomerase A) [91] and *Rpl30* (ribosomal protein L30) [92].

### 4.4. Motif Enrichment Analysis

We performed this analysis on promoters of the genes upregulated and downregulated in the brain tissue samples of tame rats. For this purpose, we developed the ESDEG tool, which is available at https://github.com/ubercomrade/esdeg (accessed on 12 October 2022). The full set of 18,442 promoter regions (−2000; +1) relative to the transcription start sites for protein-coding genes annotated in an *R. norvegicus* genome (RGSC version Rnor_6.0, UCSC, Santa Cruz, USA, version Rn6, July 2014 assembly) were employed for the analysis; alternative transcripts were disregarded. The murine core collection including 356 motifs (nucleotide frequency matrices) from the HOCOMOCO database [28] served as a list of motifs, of which only 351 motifs were included in the analysis according to a filtering approach proposed in [29]. We decided to use the murine motif collection because this species is the best-studied model organism closest to the rat with the most complete motif collection accumulated to date.

To identify overrepresented motifs, we first performed motif recognition in the whole set of 18,442 promoter regions mentioned above, utilizing each from a collection of 351 nucleotide frequency matrices. Next, a table of recognition thresholds was compiled as described in [29], where expected recognition rates (ERRs) for each threshold were calculated as probabilities of site prediction in the whole-genome dataset of promoters. Given that the enrichment of sites depends on a chosen threshold, subsequent calculations were performed for 30 recognition thresholds (equidistant on a logarithmic scale of ERRs) in the range of 5 × 10^−4^ to 1 × 10^−4^. Next, all promoters were categorized into either a foreground or background group. The foreground group contained the promoters of the significantly upregulated or downregulated genes. We utilized the following criteria to determine whether a gene was differentially expressed: an adjusted *p*-value below 0.1 and a fold change above 2.0 (alternatively: above 1.5). The background group included the same number of promoters as the foreground group, but they were randomly selected according to the following criteria: an adjusted *p*-value above 0.1 and a fold change within the range of 0.80 to 1.25. Next, we calculated the frequency of a motif in each of the gene promoter groups. To estimate the significance of the motif enrichment, we applied the Monte Carlo approach. For a given motif and a recognition threshold, site frequency was calculated for the foreground group (AV_FOR_), whereas a background group was generated many times to estimate the site frequency distribution. Next, the average (AV_BACK_) and standard deviation (SD_BACK_) for the frequencies in the background group set were calculated. Then, a Z-score was calculated according to the formula (AV_FOR_ − AV_BACK_)/SD_BACK_. This Z-score allowed us to compute the significance of enrichment (*p*-value) by fitting a site’s frequency distribution to a normal distribution. To confirm enrichment for a given motif, we combined multiple *p*-values, referring all recognition thresholds into a single unified *p*-value according to the Hartung method [93]. Thus, a unified *p*-value was calculated for each motif. Finally, we calculated an adjusted *p*-value (p_adj_) from the set of unified *p*-values for all motifs with the Benjamini–Hochberg correction for multiple comparisons; in this way, our analysis involved multiple simultaneous statistical tests for various motifs.

### 4.5. Statistical Analysis

We performed the Mann–Whitney *U* test and Fisher’s Z-test using options in the STATISTICA standard toolbox (Statsoft^TM^, Hamburg, Germany).

## 5. Conclusions

We performed RNA-seq to investigate differential expression of genes in tissue samples from the tegmental region of the midbrain of rats showing either tame or aggressive behavior. The list of DEGs includes genes related to extracellular matrix components, transporter proteins, neurotransmitter system genes, signaling molecules, and immune-system proteins, as well as genes encoding transcription factors. A new function of the ASCL3 TF, which is upregulated in the brain tissue samples of tame rats, is proposed. We demonstrated that several structural subtypes of E-box motifs—known as binding sites for many developmental TFs of the bHLH class, including the ASCL subfamily of TFs—are enriched in the set of promoters of the DEGs downregulated in the tissue samples of tame rats. We showed that the increased expression of the *Ascl3* gene in tame rats is the most likely cause of the statistically significant downregulation of the corresponding genes, as its product, ASCL3 TF, presumably acts as a repressor. In aggressive rats, the *Ascl3* gene is expressed at a low level; therefore, ASCL3 TF does not inhibit the expression of the same genes, so they are upregulated compared to tame rats. Thus, we hypothesize that the upregulation of *Ascl3* in tame rats plays an important role in changes of the behavior of animals during domestication, probably promoting longer neurogenesis (as compared to aggressive rats), which is a sign of neoteny and domestication.

## Figures and Tables

**Table 1:** Summary statistics for differentially expressed genes (DEGs) in the tegmental region of the midbrain (MT) transcriptomes of three tame adult male rats (*Rattus norvegicus*) and three aggressive rats.

Group	Count
Total number of sequence reads (NCBI SRA ID: PRJNA668014)	182,197,974
Reads mapped to reference rat genome RGSC Rnor_6.0, UCSC Rn6, July 2014 (%)	158,310,590 (86.9%)
Expressed genes identified	14,039
Statistically significant DEGs (|log_2_ fold| > 1, P_ADJ_ < 0.1, Fisher’s Z-test with Benjamini–Hochberg correction)	42

During the processing of the RNA-seq data, significant differences were revealed between the aggressive and tame strains of rats in the transcriptome profile of the MT.

**Table 2:** DEGs in the MT of tame vs. aggressive rats.

#No.	Rat Gene, Name	Symbol	log_2_ fold	P_ADJ_
1	defensin beta 17	Defb17	7.31	1.86 × 10^−3^
2	leukocyte immunoglobulin-like receptor, subfamily B member 3-like	Lilrb3l	5.71	9.15 × 10^−2^
3	lipoxygenase homology PLAT domains 1	Loxhd1	4.02	7.91 × 10^−2^
4	sucrase-isomaltase	Si	3.30	8.66 × 10^−2^
5	achaete-scute family bHLH transcription factor 3	Ascl3 *	2.82	3.16 × 10^−4^
6	protocadherin gamma subfamily A, 1	Pcdhga1	2.80	2.21 × 10^−2^
7		LOC100910802	2.25	6.64 × 10^−2^
8	Fc gamma receptor IIb	Fcgr2b	2.01	2.46 × 10^−2^
9	cysteine-rich secretory protein LCCL domain containing 1	Crispld1	1.49	7.40 × 10^−2^
10	collagen triple helix repeat containing 1	Cthrc1	1.44	6.13 × 10^−2^
11	solute carrier family 7 member 11	Slc7a11	1.37	9.84 × 10^−2^
12	nicotinamide nucleotide adenylyltransferase 1	Nmnat1	1.31	1.76 × 10^−11^
13	5-hydroxytryptamine receptor 3A	Htr3a	1.30	4.33 × 10^−2^
14	macrophage expressed 1	Mpeg1	1.28	8.30 × 10^−4^
15	schlafen family member 13	Slfn13	1.26	6.30 × 10^−2^
16	syncoilin, intermediate filament protein	Sync	1.19	5.31 × 10^−2^
17	olfactomedin-like 1	Olfml1	1.14	6.73 × 10^−2^
18	phosphotriesterase related	Pter	1.13	7.16 × 10^−2^
19	collagen type XV alpha 1 chain	Col15a1	1.12	5.45 × 10^−2^
20	MORN repeat containing 1	Morn1	1.10	1.43 × 10^−4^
21	somatostatin receptor 2	Sstr2	−1.01	2.46 × 10^−2^
22	retinol saturase	Retsat	−1.03	1.33 × 10^−2^
23	glycerol-3-phosphate dehydrogenase 1	Gpd1	−1.05	2.46 × 10^−2^
24	aquaporin 9	Aqp9	−1.22	9.89 × 10^−2^
25	serine peptidase inhibitor, Kunitz type 1	Spint1	−1.29	1.43 × 10^−4^
26	activity-regulated cytoskeleton-associated protein	Arc	−1.35	6.13 × 10^−2^
27	t-complex-associated testis expressed 1	Tcte1	−1.41	6.30 × 10^−2^
28	tetratricopeptide repeat domain 22	Ttc22	−1.44	5.08 × 10^−2^
29	keratin2	Krt2	−1.54	8.63 × 10^−5^
30	Fos proto-oncogene, AP-1 transcription factor subunit	Fos*	−1.63	6.87 × 10^−2^
31	synaptophysin-like 2	Sypl2	−1.65	8.72 × 10^−2^
32	Ly6/Plaur domain containing 3	Lypd3	−1.86	5.08 × 10^−2^
33	5-hydroxytryptamine (serotonin) receptor 5B	Htr5b	−1.94	9.17 × 10^−2^
34	FosB proto-oncogene, AP-1 transcription factor subunit	Fosb*	−1.95	2.46 × 10^−2^
35	heat shock protein family A (Hsp70) member 1A	Hspa1a	−2.10	4.25 × 10^−2^
36	heat shock protein family A (Hsp70) member 1B	Hspa1b	−2.19	3.42 × 10^−8^
37	vasoactive intestinal peptide	Vip	−2.35	8.93 × 10^−3^
38		RGD1565611	−2.94	6.69 × 10^−2^
39	tachykinin precursor 3	Tac3	−3.64	2.46 × 10^−2^
40	protein tyrosine phosphatase, non-receptor type 20	Ptpn20	−4.49	9.17 × 10^−2^
41	hypocretin neuropeptide precursor	Hcrt	−5.15	6.56 × 10^−2^
42	hemoglobin, beta adult major chain	Hbb-b1	−7.78	1.09 × 10^−5^

Note. log_2_ fold: log_2_-transformed fold change (i.e., ratio of an expression level of a given gene in tame rats to that in aggressive rats); P_ADJ_: statistical significance according to Fisher’s Z-test with Benjamini–Hochberg correction for multiple comparisons. Results are ordered by log_2_ fold change. Positive log_2_ fold change values indicate genes upregulated in tame rat samples; negative log_2_ fold change values indicate genes upregulated in aggressive rat samples. Transcription factor genes are marked with an asterisk.

**Table 3:** qPCR primers selected using the publicly available PrimerBLAST web service [89].

No.	Gene	Forward, 5′→3′	Reverse, 5′→3′
DEGs identified in the hippocampus of tame versus aggressive adult male rats [This Work]			
1	*Ascl3*	CCTCTGCTGCCCTTTTCCAG	ACTTGACTCGCTGCCTCTCT
2	*Defb17*	TGGTAGCTTGGACTTGAGGAAAGAA	TGCAGCAGTGTGTTCCAGGTC
Reference Genes			
3	*Ppia*	TTCCAGGATTCATGTGCCAG	CTTGCCATCCAGCCACTC
4	*Rpl30*	CATCTTGGCGTCTGATCTTG	TCAGAGTCTGTTTGTACCCC

Notes. Regarding the DEGs subjected to this qPCR verification, see Table 2; reference rat genes: *Ppia* (peptidylprolyl isomerase A [91]) and *Rpl30* (ribosomal protein L30 [92]).

## Data Availability

The primary RNA-Seq data obtained in this work were deposited in the NCBI SRA database (ID = PRJNA668014).

## Supplementary Materials

The following supporting information can be downloaded at: https://www.mdpi.com/article/10.3390/ijms232012269/s1.

## Abbreviations

ASCL	achaete-scute complex like
bHLH	basic helix–loop–helix
DEG	differentially expressed gene
MT	tegmental region of the midbrain
PAG	periaqueductal gray
qPCR	quantitative PCR
SNP	single-nucleotide polymorphism
TF	transcription factor
TBP	TATA-binding protein
