# Supplementary material for: Transcription Factors as Important Regulators of Changes in Behavior through Domestication of Gray Rats: Quantitative Data from RNA Sequencing
Source: Int J Mol Sci. 2022 Oct 14;23(20):12269. doi: 10.3390/ijms232012269 (PMC9603081; doi:10.3390/ijms232012269)
Supplement: Supplementary file 1 [file ijms-23-12269-s001.zip › ijms-1918211-supplementary.pdf]

Supplementary Materials

Transcription Factors as the Important Regulators of changes in behavior through domestication of gray rats: quantitative data from RNA sequencing

Dmitry Oshchepkov*, Irina Chadaeva, Rimma Kozhemyakina, Svetlana Shikhevich, Ekaterina Sharypova, Ludmila Savinkova, Natalya Klimova, Anton Tsukanov, Victor Levitsky, Arcady Markel

Institute of Cytology and Genetics, Novosibirsk 630090, Russia

*Correspondence: diman@bionet.nsc.ru.

Abstract: Studies on hereditary fixation of the tame-behavior phenotype during animal domestication remain relevant and important because they have both basic-research and applied significance. In model animals, gray rats *Rattus norvegicus* bred for either an enhancement or reduction of a defensive response to humans, we for the first time used high-throughput RNA sequencing to investigate differential expression of genes in tissue samples from the tegmental region of the midbrain in 2-month-old rats showing either tame or aggressive behavior. Forty-two differentially expressed genes (DEGs, adjusted P-value < 0.01 and fold-change > 2) were identified in total, with 20 upregulated and 22 downregulated genes in the tissue samples from tame rats compared with aggressive ones. Among them, three genes encoding transcription factors (TFs) were detected: *Ascl3* is upregulated while *Fos* and *Fosb* are downregulated in tame rats' brain tissue samples. Other DEGs were annotated as associated with extracellular-matrix components, transporter proteins, neurotransmitter system, signaling molecules, and immune-system proteins. We believe that these DEGs encode proteins that constitute a multifactorial system determining the behavior for which the rats have been artificially selected. We demonstrated that several structural subtypes of E-box motifs—known as binding sites for many developmental TFs of the bHLH family, including the ASCL subfamily of TFs—are enriched in the set of promoters of the DEGs downregulated in tame rats' tissue samples. Because ASCL3 may act as a repressor on target genes of other developmental TFs of the bHLH family, we can hypothesize that the expression of TF gene *Ascl3* in tame rats indicates longer neurogenesis (as compared to aggressive rats), which is a sign of neoteny and domestication. Thus, our domestication model shows a new function of TF ASCL3: it may play the most important role in behavioral changes in animals.

CONTENT

Table S1. Extended list of DEGs in the tegmental of the midbrain of tame rats vs. aggressive rats, all genes with P_{ADJ}.<0.1.

Figure S1. The ranking and classification of the overrepresented motifs of known TFs in DEGs, selected according to criteria of $P_{ADJ} < 0.01$ and fold-change > 1.5 (log_2 fold >0.585).

S1 Supplementary methods for *in silico* comparing of two promoter DNA sequences in estimates of affinity for TBP protein

Figure S2. The illustrative example of the result produced by the Web-service Human_SNP_TATA_Z-tester in the case of the candidate SNP marker (rs1049743008) reducing the estimate of TBP-binding affinity for the proximal promoter of the human *ASCL3* gene homologous to the rat

Ascl3 gene identified in this work as the differentially expressed genes (DEGs) of tame rats compared with aggressive rats

Table S2. The *in silico* estimates and *in vitro measurements* for the -ln(K_D) values of the TBP-promoter affinity expressed in the natural logarithm units, according to all SNPs of the TBP-binding regions of the human genes *HBB*, *HBD* and *ASCL3* homologous to the rat genes *Hbb-b1* and *Ascl3* identified as the differentially expressed genes (DEGs) in tame rats compared with aggressive rats in this work.

S2. Supplementary methods for *in vitro* measurements

Figure S3. Electrophoregrams for the wild type (a) and minor (b) alleles of SNP rs1049743008 in the promoter of human *ASCL3* gene

Table S1. Extended list of DEGs in the tegmental of the midbrain of tame rats *vs.* aggressive rats, all genes with P_{ADJ}.<0.1. Transcription factor genes are marked with an asterisk.

	id	$\log_2$ fold	p-value	P_{ADJ}
1	*Defb17*	7.31	1.38E-06	1.86E-03
2	*Lilrb3l*	5.71	4.76E-04	9.15E-02
3	*Loxhd1*	4.02	3.46E-04	7.91E-02
4	*Si*	3.30	3.99E-04	8.66E-02
5	*Ascl3**	2.82	1.88E-07	3.16E-04
6	*Pcdhga1*	2.80	3.28E-05	2.21E-02
7	*LOC100910802*	2.25	2.27E-04	6.64E-02
8	*Fcgr2b*	2.01	4.03E-05	2.46E-02
9	*Crispld1*	1.49	3.08E-04	7.40E-02
10	*Cthrc1*	1.44	1.87E-04	6.13E-02
11	*Slc7a11*	1.37	5.83E-04	9.84E-02
12	*Nmnat1*	1.31	1.31E-15	1.76E-11
13	*Htr3a*	1.30	1.03E-04	4.33E-02
14	*Mpeg1*	1.28	5.55E-07	8.30E-04
15	*Slfn13*	1.26	2.06E-04	6.30E-02
16	*Sync*	1.19	1.50E-04	5.31E-02
17	*Olfml1*	1.14	2.45E-04	6.73E-02
18	*Pter*	1.13	2.82E-04	7.16E-02

19	*Col15a1*	1.12	1.58E-04	5.45E-02
20	*Morn1*	1.10	7.42E-08	1.43E-04
21	*LOC500475*	0.97	1.44E-05	1.33E-02
22	*Cd48*	0.96	6.05E-04	9.85E-02
23	*Tecta*	0.93	1.68E-05	1.33E-02
24	*Rbm3*	0.92	7.54E-06	8.46E-03
25	*LOC100366030*	0.92	4.86E-04	9.17E-02
26	*Cyp2j10*	0.91	9.54E-05	4.25E-02
27	*Bcl2l11*	0.88	4.47E-04	8.72E-02
28	*Irf6**	0.87	1.37E-04	5.08E-02
29	*Scd1*	0.86	1.66E-04	5.57E-02
30	*Pdpn*	0.78	5.67E-04	9.79E-02
31	*Zfp26*	0.70	2.60E-04	6.87E-02
32	*Lyn*	0.67	6.25E-05	3.12E-02
33	*Bdh1*	0.44	4.41E-06	5.40E-03
34	*Pygm*	-0.39	2.72E-04	7.05E-02
35	*Pja1*	-0.40	3.21E-04	7.56E-02
36	*Tmed4*	-0.42	6.07E-04	9.85E-02
37	*Pdia4*	-0.49	3.06E-04	7.40E-02
38	*Spire2*	-0.50	5.12E-04	9.19E-02
39	*Capns1*	-0.51	4.42E-04	8.72E-02
40	*Tril*	-0.55	5.46E-04	9.54E-02
41	*Xbp1**	-0.56	1.31E-04	5.08E-02
42	*Cln6*	-0.57	4.36E-04	8.72E-02
43	*Doc2a*	-0.57	5.18E-04	9.19E-02
44	*Banp**	-0.59	2.44E-04	6.73E-02
45	*Paqr4*	-0.62	5.19E-04	9.19E-02
46	*Nt5c3b*	-0.63	1.92E-05	1.43E-02
47	*Fam212b*	-0.63	1.40E-04	5.08E-02

48	*Tmod1*	-0.65	3.73E-04	8.37E-02
49	*RGD735029*	-0.66	5.87E-04	9.84E-02
50	*Plod1*	-0.69	9.78E-05	4.25E-02
51	*Mrap2*	-0.70	4.40E-04	8.72E-02
52	*Dnajb1*	-0.70	5.92E-04	9.84E-02
53	*Dok5*	-0.71	4.08E-04	8.72E-02
54	*Nfil3**	-0.71	3.86E-04	8.51E-02
55	*Vstm2b*	-0.72	8.50E-05	4.09E-02
56	*Rtn4ip1*	-0.79	2.84E-05	2.02E-02
57	*Spry4*	-0.80	1.57E-05	1.33E-02
58	*Mcm7**	-0.85	4.63E-05	2.46E-02
59	*Actn2*	-0.86	3.26E-04	7.56E-02
60	*Txnrd2*	-0.92	4.29E-10	1.93E-06
61	*Cys1*	-0.93	4.32E-04	8.72E-02
62	*Mblac1*	-0.99	2.89E-04	7.22E-02
63	*Sstr2*	-1.01	4.51E-05	2.46E-02
64	*Retsat*	-1.03	1.58E-05	1.33E-02
65	*Gpd1*	-1.05	4.74E-05	2.46E-02
66	*Aqp9*	-1.22	6.17E-04	9.89E-02
67	*Spint1*	-1.29	7.09E-08	1.43E-04
68	*Arc*	-1.35	1.91E-04	6.13E-02
69	*Tcte1*	-1.41	2.06E-04	6.30E-02
70	*Ttc22*	-1.44	1.39E-04	5.08E-02
71	*Krt2*	-1.54	3.20E-08	8.63E-05
72	*Fos**	-1.63	2.59E-04	6.87E-02
73	*Sypl2*	-1.65	4.42E-04	8.72E-02
74	*Lypd3*	-1.86	1.27E-04	5.08E-02
75	*Htr5b*	-1.94	4.97E-04	9.17E-02
76	*Fosb**	-1.95	4.39E-05	2.46E-02

77	*Hspa1a*	-2.10	9.29E-05	4.25E-02
78	*Hspa1b*	-2.19	5.08E-12	3.42E-08
79	*Vip*	-2.35	8.62E-06	8.93E-03
80	*RGD1565611*	-2.94	2.34E-04	6.69E-02
81	*Tac3*	-3.64	4.36E-05	2.46E-02
82	*Ptpn20*	-4.49	4.96E-04	9.17E-02
83	*Hcrt*	-5.15	2.19E-04	6.56E-02
84	*Hbb-b1*	-7.78	3.23E-09	1.09E-05

A

Rank	Enrichment significance, -Log10(padj)	TF family	Hocomoco ID	BRAC_MOUSE.H11MO.0.B	TBX21_MOUSE.H11MO.0.A	PO2F1_MOUSE.H11MO.0.C	REL_MOUSE.H11MO.0.A	ARI5B_MOUSE.H11MO.0.C	TF65_MOUSE.H11MO.0.A	AIRE_MOUSE.H11MO.0.C	FOSL2_MOUSE.H11MO.0.A	PO2F2_MOUSE.H11MO.0.B	CTCF_MOUSE.H11MO.0.A
1	235.1	Brachyury-related factors{6.5.1}	BRAC_MOUSE.H11MO.0.B										
2	121.2	TBrain-related factors{6.5.2}	TBX21_MOUSE.H11MO.0.A	3.1									
3	35.5	POU domain factors{3.1.10}	PO2F1_MOUSE.H11MO.0.C										
4	2.7	NF-kappaB-related factors{6.1.1}	REL_MOUSE.H11MO.0.A										
5	2.7	ARID-related factors{3.7.1}	ARI5B_MOUSE.H11MO.0.C										
6	2.7	NF-kappaB-related factors{6.1.1}	TF65_MOUSE.H11MO.0.A				6.8						
7	2.4	AIRE{5.3.1}	AIRE_MOUSE.H11MO.0.C										
8	2.3	Fos-related factors{1.1.2}	FOSL2_MOUSE.H11MO.0.A										
9	1.6	POU domain factors{3.1.10}	PO2F2_MOUSE.H11MO.0.B			3.3							
10	1.4	More than 3 adjacent zinc finger factors{2.3.3}	CTCF_MOUSE.H11MO.0.A										

B

Rank	Enrichment significance, -Log10(padj)	TF family	Hocomoco ID	IRF3_MOUSE.H11MO.0.A	SPIB_MOUSE.H11MO.0.A
1	48.9	Interferon-regulatory factors{3.5.3}	IRF3_MOUSE.H11MO.0.A		
2	1.8	Ets-related factors{3.5.2}	SPIB_MOUSE.H11MO.0.A		

Figure S1. The ranking and classification of the overrepresented motifs of known TFs in DEGs, selected according to criteria of $P_{ADJ} < 0.01$ and fold-change > 1.5 ($\log_2$ fold > 0.585). Panels A/B show 10/2 top-scored motifs detected for the promoters of genes, up- and down-regulated in tame samples, respectively (see Table 2). Motifs overrepresentation were estimated by the ESDEG tool (see Materials and Methods). Motifs of known TFs were compiled from the mouse core collection of the Hocomoco database (see Materials and Methods). On the left, each panel shows four columns indicating the ranks of motifs, the significance of their enrichment computed by ESDEG ($-\text{Log}_{10}(p_{adj})$), the respective TF families according to the classification of murine TFs based on their DNA-binding domains [17], and

the names of TFs (Hocomoco ID). Green color in the panel A marks the motifs for FOSL2_MOUSE.H11MO.0.A from the family Fos-related factors{1.1.2}, this motif is similar to known motifs of TFs from the same family FOS/FOSB, which were revealed in the list of DEGs above (Table 2). On the right, each panel presents the lower part of the symmetrical matrix depicting the pairwise similarities of motifs that were estimated by the permutation test described earlier [29]. In particular, the empty cells imply significantly distinct motifs (p-value > 0.05), the shades from light orange to dark orange mark the significantly similar motifs (p-value < 0.05), and the numbers designate the common logarithm of the significance, $-\text{Log}_{10}$(p-value). To simplify the visualization, we retained only the values and shading only in the bottom-left corner of the similarity matrix.

S1 Supplementary methods for *in silico* comparing of two promoter DNA sequences in estimates of affinity for TBP protein

We used our previously created Web service Human_SNP_TATA_Z-tester [53] (see Figure S2) for the calculations. Its input data include two 90 bp proximal promoter DNA sequences, $S_{wt} = \{s^{wt}_{-90}\ldots s^{wt}_{i}\ldots s^{wt}_{-1}\}$ and $S_{m} = \{s^{min}_{-90}\ldots s^{min}_{i}\ldots s^{min}_{-1}\}$. These sequences are located immediately upstream of the transcription start site (TSS, $s^{wt}_{0} = s^{min}_{0}$, where: $s^{\bullet}_{i} \in \{a, c, g, t\}$) of the human gene carrying either ancestral (wt) or minor (min) allele, respectively, of an arbitrary SNP under study. The figure shows these input data: two textboxes "1st promoter" and "2nd promoter", respectively. Using SNP rs1049743008 as an illustrative example, the double dashed arrows in Figures S2a and S2b show how one can take these two DNA sequences using the UCSC Genome Browser [54] and the dbSNP database [51], respectively.

Our Web service utilizes previously experimentally proved three-step model [55,56] of the TBP-promoter binding (i.e., TBP slides along DNA [57] ↔ mutual molecular recognition between TBP and a potential TBP-site encountered [58] ↔ DNA-bend stabilizes TBP-promoter complex [59]) . Upon picking the "Calculate" button (Figure S2c), our Web-service [53] estimates two "$-\ln[K_D(S_\bullet)]$"-values expressed on the natural-logarithm scale (ln-units), which rates the TBP-promoter affinity upon each DNA sequence ($S_\bullet$) independently one from another, namely:

$$-\ln[K_D(S_\bullet)] = 10.9 - 0.2\,\{\ln[K_{SLIDE}(S_\bullet)K_{STOP}(S_\bullet)K_{BEND}(S_\bullet)]\}, \qquad \textbf{(S1)}$$

where K_D is the equilibrium dissociation constant evaluation (in moles per liter, M); the value 10.9 (ln-units) fits numerically to nonspecific TBP–DNA affinity (10 μM) as were identified independently [60]; the value 0.2 is the stoichiometric coefficient of the three-step TBP–promoter binding, as suggested elsewhere according to the difference in the length of the TBP binding site and the region of TBP sliding along DNA [55].

Within the Eq. S1, $-\ln[K_{STOP}(S_\bullet)]$ is the evaluation of the equilibrium dissociation constant of the mutual molecular recognition between TBP and the most probable TBP-site met at the second step within the three-step approximation of the TBP-promoter complex formation molecular mechanism [55], such as:

$$-\ln[K_{STOP}(S_\bullet)] = \text{MAX}_{-90 \le i \le -20;\ k \in \{-1;+1\}}\{\Sigma_{\,i-1 \le j \le i+13}\ w\{i, s^{\bullet}_{j;k}\}, \qquad \textbf{(S2)}$$

where $w\{i, s^{\bullet}_j\}$ is the Bucher's weight of nucleotide $s^{\bullet}_j$ at the *j*th position of the TBP-site [61]; k is an indicator of either the direct (+1) or reverse(-1) strand of DNA of a promoter under study.; MAX(ζ) is the highest ζ-value observed.

Additionally, in the Eq. S1, $-\ln[K_{SLIDE}(S\bullet)]$ is the evaluation of the equilibrium dissociation constant of the interaction between TBP and the promoter DNA during their sliding one over the other at the first step among the three steps in question [55], as follows:

$$-\ln[K_{SLIDE}(S\bullet)] = MEAN_{[\xi - 7;\, \xi + 19];\ k \in \{-1;\, +1\}}\,(35.1\mu + 0.8[TA]), \qquad \textbf{(S3)}$$

where ξ is the position of the most probable TBP-site by means of the Bucher's criterion [61] (i.e., Eq. S2); the value μ of the minor-groove width of the B-helical DNA at this site's center was determined elsewhere [62]; [TA] is the amount of dinucleotide TA; 0.8 and 35.1 are linear regression coefficients [63].

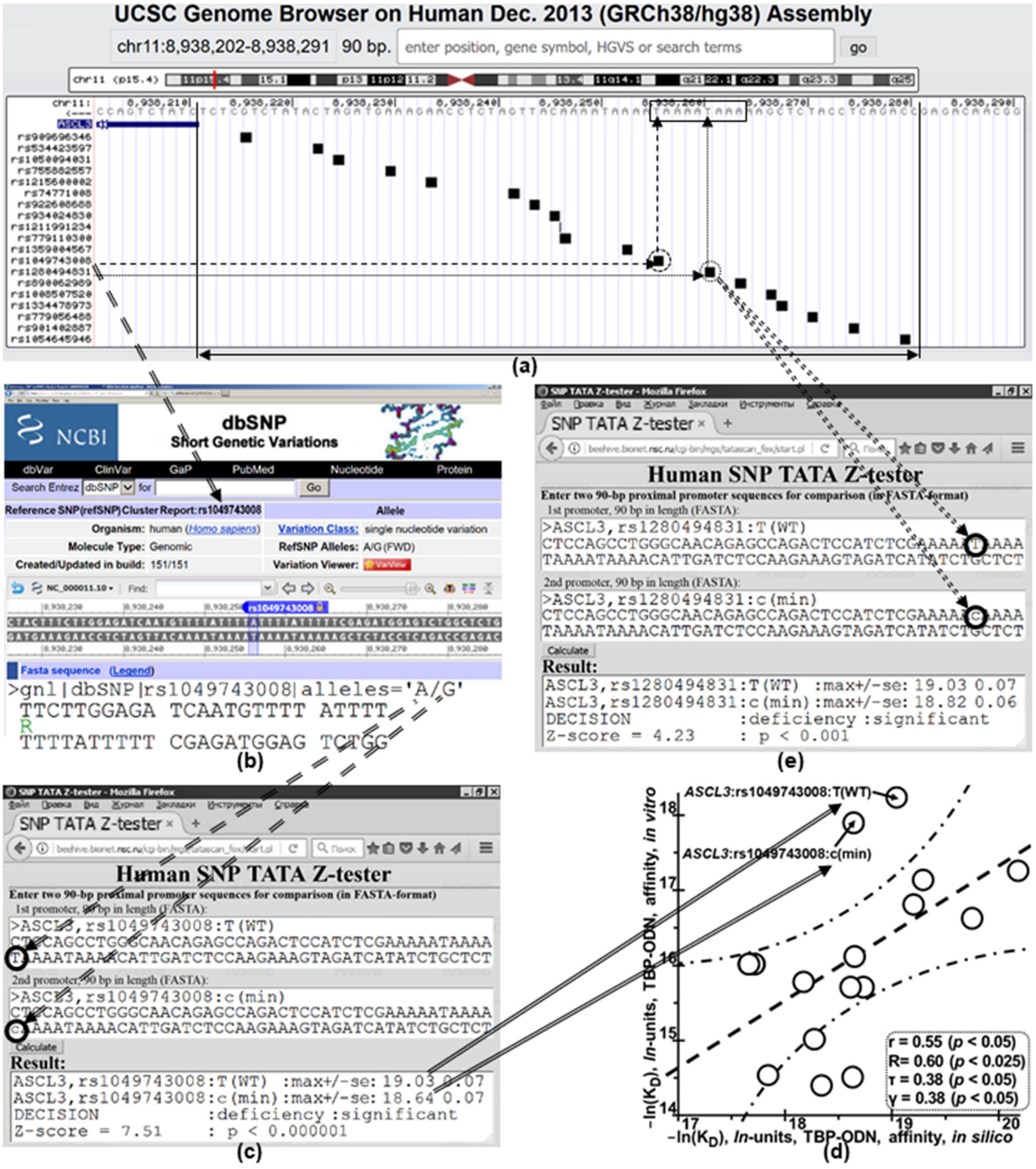

Figure S2. The illustrative example of the result produced by the Web-service Human_SNP_TATA_Z-tester [53] in the case of the candidate SNP marker (rs1049743008) reducing the estimate of TBP-binding affinity for the proximal promoter of the human *ASCL3* gene homologous to the rat *Ascl3* gene identified in this work as the differentially expressed genes (DEGs) of tame rats compared with aggressive rats. Legend: **a)** The UCSC Genome Browser [54] visualizes the promoter region in question (double-headed arrow) of the human *ASCL3* gene in question with only one TBP-binding site (framed) and two SNPs rs1049743008 and rs1280494831 with the arrows pointing to them; black squares, all 18 SNPs documented within the dbSNP rel. 151 [51]; two circles, two SNPs rs34166473 and rs1280494831 reducing the TBP-promoter affinity according to the result of the Web-service Human_SNP_TATA_Z-

tester [53 (**c** and **e**, respectively)]. **b)** the dbSNP database describes the SNP under study (rs1049743008 in this example). Double dashed arrows depict how one can input data on the SNPs (e.g. rs1049743008) into the Web-service Human_SNP_TATA_Z-tester [53]. **d)** statistically significant correlations between the *in silico* predicted and *in vitro* measured "-ln(K_D)-values of the TBP-promoter affinity dependent on SNPs listed in Table S2. Dashed and dash-and-dot lines denote the linear regression and boundaries of its 95% confidence interval. Notations r, R, τ, and p mean coefficients of Pearson's linear correlation, Spearman's rank correlation, Kendall's rank correlation, and their statistical significance (p values), respectively.

Besides, in the Eq. S1, $-\ln[K_{BEND}(S_\bullet)]$ is the evaluation of the equilibrium dissociation constant of intermediate short-lived complexes between TBP and each of the two DNA strands of the TBP-site (separately one from another) during the DNA melting, which leads to the bend that fixes the TBP–promoter complex [59] at the last step of the binding, as:

$$-\ln[K_{BEND}(S_\bullet)] = \mathrm{MEAN}_{[\xi-7;\xi+19];\ k\hat{I}\{-1;+1\}}\,(0.9[TA, AA, TG, AG] + 2.5[TA, TC, TG] + 14.4), \qquad \textbf{(S4)}$$

where 0.9, 2.5, and 14.4 are linear regression coefficients [63].

Thereafter, examining all the possible mutations, $s^\bullet_j \rightarrow \varphi$, at each *j*th position among 26 positions of the most probable TBP-site according to the Eq. S2, the Web-service [53] rates the standard error SE● of the $-\ln[K_D(S_\bullet)]$ values calculated using Eq. S1, as:

$$SE_\bullet=\{(\Sigma_{\xi-7 \le j \le \xi+19}\Sigma_{\varphi\ \{a,c,g,t\}}\ln[K_D(s^\bullet_{\xi-7}\ldots s_{j-1}\varphi s_{j+1}\ldots s^\bullet_{\xi+19}) / K_D(s^\bullet_{\xi-7}\ldots s_{j-1}s_j\, s_{j+1}\ldots s^\bullet_{\xi+19})]^2\} / ((3*26)(3*26 - 1))]^{1/2}. \quad \textbf{(S5)}$$

Using both sequences S_{wt} and S_{min} and Eqs. S1 - S5, this toolbox find two paired value sets $\{-\ln[K_D(S_{wt})] \pm SE_{wt}\}$ and $\{-\ln(K_D(S_{min})) \pm SE_{min}\}$, respectively, which are necessary for Fisher's Z-score [64], for instance:

$$Z = \mathrm{abs}\{\ln[K_D(S_{wt}) / K_D(S_m))] / [SE_{wt}^2 + SE_m^2\}^{1/2}.. \qquad \textbf{(S6)}$$

Finally, by means the R software package [64], using this Z-value, our Web service Human_SNP_TATA_Z-tester [53] finds the *p* value of the probability of the tested hypothesis "H_0: $K_D(S_{wt}) = K_D(S_{min})$" , in case it is statistically significant ($p < 0.05$), it makes the decision:

IF {**INEQUALITY** "$K_D(S_{min}) < K_D(S_{wt})$" is statistically significant },

THEN {**PREDICTION** is "the minor allele of the gene considered is overexpressed relative to the ancestral one"};

ELSE [**IF** {**INEQUALITY** "$K_D(S_m) > K_D(S_{wt})$" is statistically significant}, **(S7)**

THEN {**DECISION** is "the minor allele of this gene is underexpressed relative to the ancestral one"},]

OTHERWISE {**DECISION** is "the expression change of this gene is insignificant"}.

In the "Decision" line of the "Result" textbox (Figure S2c), one can see this decision (Eq. S7) made by the Web service Human_SNP_TATA_Z-tester [53] together with all the intermediate results, which are shown at the other lines of this textbox. The column "*in silico*" of Table S2 presents the estimates for 16 among all 17 SNPs, which can significantly alter the TBP-binding affinity. Estimates for the human genes *HBB*, *HBD* and *ASCL3* homologous to the rat genes *Hbb-b1* and *Ascl3* are shown. These genes were identified as the differentially expressed genes (DEGs) of tame rats compared with aggressive rats in this work (Table2).

Table S2. The *in silico* estimates and *in vitro measurements* for the -ln(K_D) values of the TBP-promoter affinity expressed in the natural logarithm units, according to all SNPs of the TBP-binding regions of the human genes *HBB, HBD* and *ASCL3* homologous to the rat genes *Hbb-b1* and *Ascl3* identified as the differentially expressed genes (DEGs) in tame rats compared with aggressive rats in this work.

#	***Human gene,* dbSNP ID [Day, 2010]**	**Allele**	**26 bp oligodeoxyribonucleotides, direct strand of the promoter**	*in silico*	*in vitro*
1	*HBB*	WT	cagggctggg**<u>C</u>**A**<u>TAAAA</u>**gtcagggca	19.20	16.81
2	rs34598529	A-28G	cagggctgggCATA**<u>G</u>**AAgtcagggca	18.34	14,40
3	rs34598529	A-28C	cagggctgggCATA**<u>C</u>**AAgtcagggca	18.63	14.51
4	rs281864525	A-25C	cagggctgggCATAAA**<u>C</u>**gtcagggca	18.73	15.71
5	rs63750953	Δ-25AA	ccagggctgggCATA**<u>*Ag*</u>**tcagggcag	18.61	15.71
6	rs33980857	T-29A	cagggctgggCA**<u>A</u>**AAAAgtcagggca	17.70	16.02
7	rs33980857	T-29C	cagggctgggCA**<u>C</u>**AAAAgtcagggca	18.17	15.78
8	rs33980857	T-29G	cagggctgggCA**<u>G</u>**AAAAgtcagggca	17.67	16.02
9	rs33931746	A-27T	cagggctgggCATA**<u>T</u>**AAgtcagggca	19.75	16.63
10	rs34598529	A-28G	cagggctgggCAT**<u>G</u>**AAAgtcagggca	17.85	14.51

11	rs34500389	C-32T	cagggctggg**T**ATAAAAgtcagggca	20.18	17.26
12	*HBD*	WT	acaggaccagC**AT**AAAAggcagggca	19.29	16.47
13	rs35518301	A-31G	acaggaccagC**G**TAAAAggcagggca	18.65	16.12
14	rs34166473	T-30C	acaggaccagCA**T**AAAAggcagggca	18.27	15.59
15	*ASCL3*	WT	tcgaaaaa**T**AAAA**T**AAAAtaaaacat	19.03	15.05
16	rs1049743008	T-45c	tcgaaaaa**T**AAAA**c**AAAAtaaaacat	18.64	16.22
17	rs1280494831	T-50c	tcgaaaaa**c**AAAA**T**AAAAtaaaacat	18.82	-

Note. Human genes: *ASCL3*, Achaete-Scute family bHLH transcription factor 3; *HBB* and *HBD*, hemoglobin subunits β and δ, respectively.

S2. Supplementary methods for *in vitro* measurements

Recombinant full-length human TBP was expressed in *Escherichia coli* BL21 (DE3) cells transformed with the pAR3038-TBP plasmid (a kind gift from Prof. B. Pugh, Pennsylvania State University) by the previously described method [65] with two modifications: the IPTG concentration was 1.0 mM instead of 0.1 mM; the induction time was 3 h instead of 1.5 h. For details of the protocol for the production and purification of human TBP, see Ref. [66]. Sixteen oligodeoxyribonucleotides (ODNs) 26 bp in length listed in Table S2 (##1-17) were synthesized by the Biosynthesis Enterprise (Novosibirsk, Russia) and were purified by PAGE. The labeled double-stranded ODNs were prepared by ^{32}P labeling of both strands by means of T4 polynucleotide kinase (SibEnzyme, Novosibirsk) with subsequent annealing by heating to 95°C (at equimolar concentrations) and slow cooling (no less than 3 h) to room temperature. The duplexes were analyzed in a 15% nondenaturing polyacrylamide gel (1 ´ Tris-borate-EDTA buffer) and isolated by electroelution. See details of the protocol for labeling of ODNs with ^{32}P in [66]. The equilibrium dissociation constants (K_D) were determined for the complexes of TBP with each 26-bp ODN in question. Experiments on association kinetics were conducted at four ODN concentrations (Figure S3).

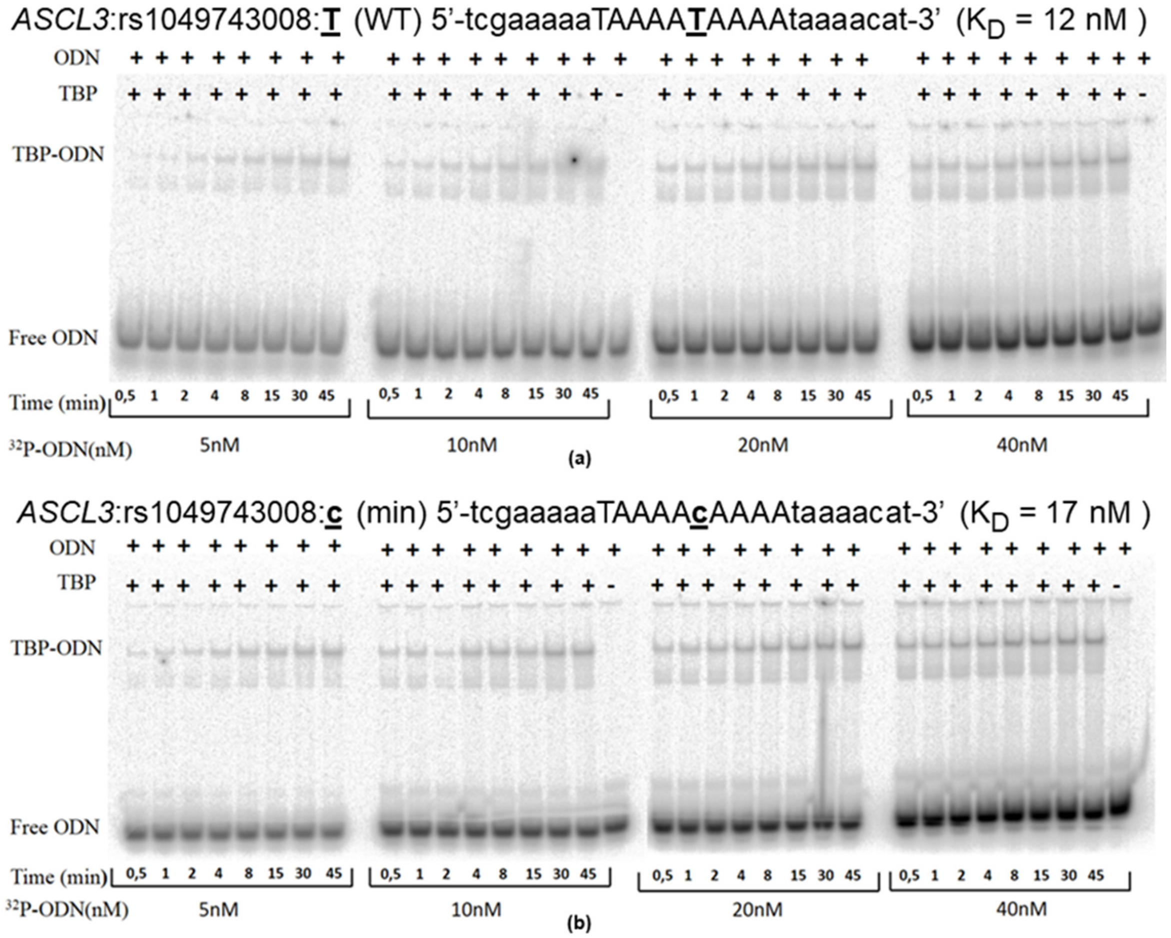

Figure S3. Electrophoregrams for the wild type (a) and minor (b) alleles of SNP rs1049743008 in the promoter of human *ASCL3* gene. The concentration of TBP was 0.3 nM in all experiments. Concentrations of 26 bp ODNs are indicated. K_D was inferred from these electropherograms using the publicly available software GraphPad Prism 5 (http://graphpad-prism.software.informer.com/5.01). The substitution T to c is indicated with bold and underlined.

The experiments with TBP–ODN binding were carried out at 25°C in binding buffer (20 mM 4-[2-hydroxyethyl-1-piperazineethanesulfonic acid [HEPES-KOH pH 7.6, 5 mM MgCl2, 70 mM KCl, 1 mM dithiothreitol (DTT), 100 μg/mL BSA, 0.01% of NP-40, and 5% of glycerol) at a fixed concentration (0.3 nM) of active TBP. The gels were dried, and Imaging Screen-K (Kodak, Rochester, NY, USA) was exposed to these gels for analysis on a Molecular Imager PharosFX Plus phosphorimager (Bio-Rad, Herts, UK). The resulting autoradiographs were quantitated in the Quantity One 4.5.0 software (Bio-Rad). Using these data as the input for publicly available software Graph-Pad Prism 5 (http://graphpad-prism.software.informer.com/5.01), we calculated the equilibrium dissociation constant (K_D). Details of the protocol for *in vitro* measurements of the equilibrium dissociation constant for TBP–ODN complexes are available elsewhere [67]. The column "in vitro" of Table S2 presents the measurements for 16 among all 17 SNPs, which can significantly alter the TBP-binding affinity estimates for the human genes *HBB*, *HBD* and *ASCL3* homologous to those for rat *Hbb-b1* and *Ascl3*, which are differentially expressed genes (DEGs) in tame rats compared with aggressive rats. Figure S2e shows statistically

significant correlations between the *in silico* predicted *and in vitro* measured -ln(K_D) values of the TBP-promoter affinity dependent on SNPs listed in Table S2.
